# Scorpions trapped in amber: a remarkable window on their evolution over time from the Mesozoic period to present days

**DOI:** 10.1590/1678-9199-JVATITD-2023-0040

**Published:** 2023-08-14

**Authors:** Wilson R. Lourenço

**Affiliations:** 1 Sorbonne Universités, Institut de Systématique, Muséum national d’Histoire naturelle, Evolution, Biodiversité (ISYEB), Paris, France.

**Keywords:** Scorpion, Fossil, Amber, Cenozoic, Baltic, Cretaceous, Burmite

## Abstract

This synoptic review aims to bring some general information on fossil scorpions, namely those trapped in amber - fossilized resin - ranging from Lower Cretaceous through the Palaeocene and up to the Miocene. The question to be addressed is how the study of these fossils can be connected with possible present scorpionism problems. A precise knowledge of these ancient lineages provides information about the evolution of extant lineages, including the buthoids, which contain most known noxious species. Among the Arthropods found trapped in amber, scorpions are considered rare. A limited number of elements have been described from the Late Tertiary Dominican and Mexican amber, while the most ancient Tertiary amber from the Baltic region produced more consistent results in the last 30 years, primarily focusing on a single limited lineage. Contrarily, the Cretaceous amber from Myanmar, also called Burmite, has yielded and continues to yield a significant number of results represented by several distinct lineages, which attest to the considerable degree of diversity that existed in the Burmese amber-producing forests. As in my previous similar contributions to this journal, the content of this note is primarily addressed to non-specialists whose research embraces scorpions in various fields such as venom toxins and public health. An overview knowledge of at least some fossil lineages can eventually help to clarify why some extant elements associated with the buthoids represent dangerous species while others are not noxious.

## Background

In a series of previous publications addressed to the readers of the *Journal of Venomous Animals and Toxins including Tropical Diseases* (JVATiTD), I attempted to bring general information about scorpions and scorpionism, but also on models of reproduction and in particular parthenogenesis [1, 2, 3]. Even some notions about how to proceed with systematics and taxonomy were the subject of one article [4]. My cooperation with the JVATiTD started with its first volume produced in 1995 [1] and continued in the following years. All the proposed contributions and, in particular, the reviews were globally addressed to non-specialists whose research embraces scorpions in several fields, such as venom toxins and public health [1, 5, 6, 7, 8, 9, 10, 11]. Most of the information previously supplied concerned historical aspects of scorpion studies but also several questions about their taxonomy, evolution, and geographic distribution [1, 5, 6, 7, 8, 9, 10, 11]. This present review aims to synthesize our knowledge about fossil scorpions trapped in amber-fossilized resin. These fossils represent a most interesting source of information about the scorpion fauna that dominated some regions of the earth during the Early Mesozoic and from the Middle to more recent Cenozoic. One question that can always be addressed is how the knowledge of these fossil scorpions can support modern studies on scorpions and scorpionism. The answer is generally simple: A precise knowledge of these ancient lineages brings elements of information about the evolution of extant lineages, including that of buthoids which contain the majority of the known noxious species.

The main target of this synopsis is once again to raise awareness among non-specialists who study scorpions in several fields such as venom toxins and public health. This is primarily because such information, when available, is typically confined to highly specialized literature, making it scattered and inaccessible to non-experts in the field. Therefore, a new presentation would be highly valuable to a wide audience. Nevertheless, some replies are also addressed to authors who recently produced synthesis and/or revisions undertaken without a comprehensive understanding of the majority of previously described taxa. Several of these decisions lead to speculative and erroneous conclusions [12, 13, 14]. These points will be better addressed in a subsequent section with taxonomic comments.

The synthesis presented in this note is mainly based on my research on fossil scorpions performed for almost 30 years now. This positive opportunity led me to describe the majority of the known taxa from amber; in some cases, almost 100%, as for the Baltic amber specimens, or more than 80% for the Burmite specimens. It must, however, be considered incomplete, since a global knowledge of all amber fossil scorpions certainly contains gaps. For some extremely rare groups, no data are presently available. But in all cases, the proposition of a more concise synopsis appears valid concerning the non-expert readers of the journal.

## General presentation

Among the fossil arthropods found in amber, scorpions remain extremely rare. The re-emergence of studies on scorpions trapped in amber started in the early 1980s when a few specimens were described from Dominican and Mexican amber [15, 16, 17, 18, 19]. Even if new taxa from Dominican and Mexican amber are yet to be described, the amber fossils found in these regions of the world seem in all cases closely related to the extant scorpion taxa of the Caribbean and North/Central Americas.

Baltic amber was the first to provide fossil scorpions, and this since the beginning of the 19^th^ century. The first species to be described was *Scorpio schweiggeri* Holl, 1829 [20]. However, both the description and illustration of this species lack accuracy; the only conclusion that can be reached is that the scorpion most certainly belongs to the family Buthidae C. L. Koch 1837. The new species described has been ignored by most authors, although Schawaller [15] produced a brief comment suggesting that *S. schweiggeri* should be considered a *nomen nudum*. Since the type specimen has been lost, not much can be added regarding its status.

**Figure 1.**
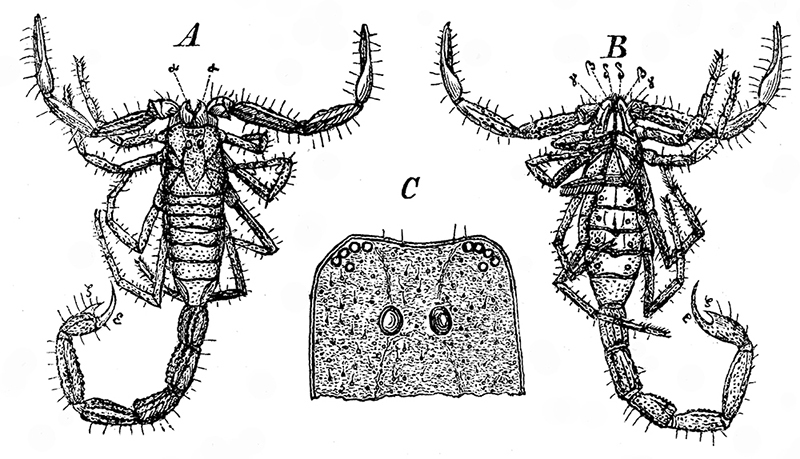
Original illustrations of *Tityus eogenus*, showing dorsal and ventral aspects (from Menge-1869) [21].

A second species, also described from Baltic amber, was *Tityus eogenus* Menge, 1869 (Fig. 1). Unlike *Scorpio schweiggeri, Tityus eogenus* has received the attention of many authors, first because of its assignment by Menge to a typically Neotropical extant genus, and secondly, because the type-specimen was seemingly lost soon after its description preventing confirmation of its taxonomic position. Menge’s collection included 2 specimens, but seemingly only one was sufficiently well preserved to be of scientific value [21, 22]. Based on this it can only be concluded that *Tityus eogenus* is indeed a buthid scorpion. It could, however, be assigned equally well to any of several genera within this family. Because of the early disappearance of Menge’s material, this Baltic amber fossil has for more than a hundred years been the subject of discussion and speculation and has been cited in several publications [22, 23, 24, 25, 26, 27].

**Figure 2.**
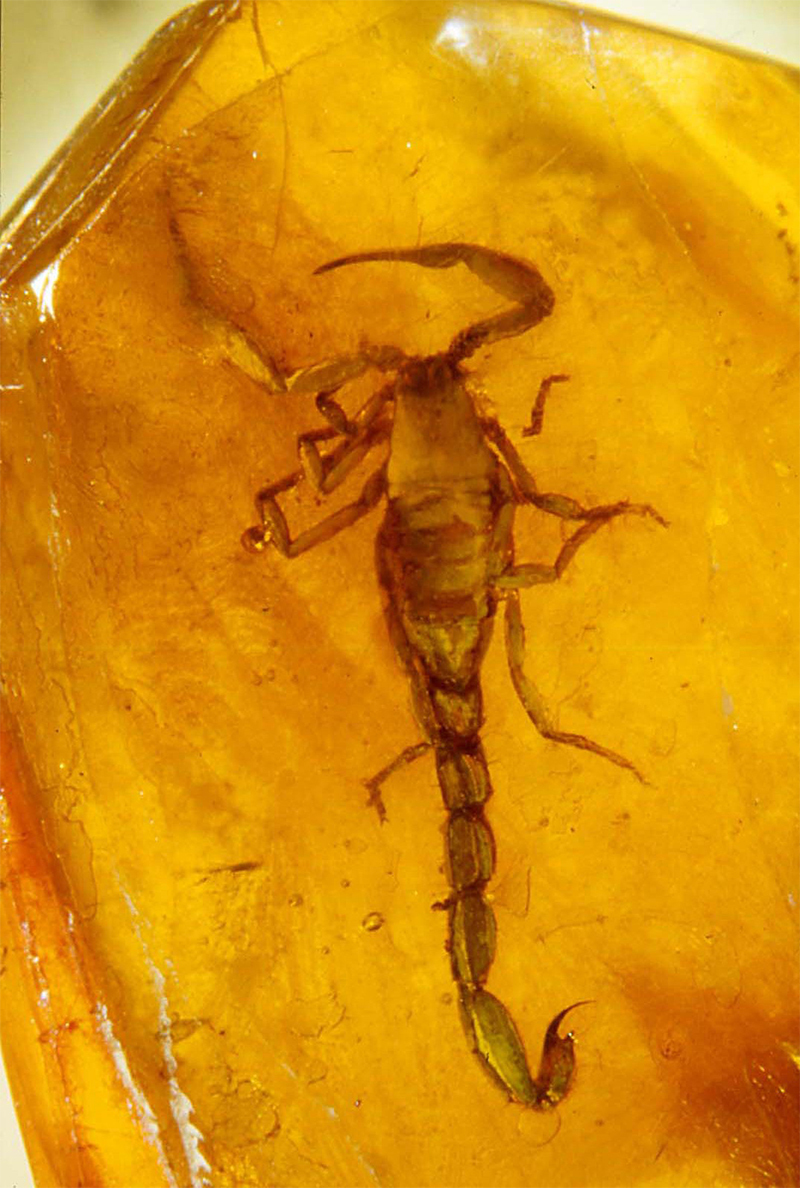
*Palaeolychas balticus*, holotype, dorsal aspect (photo, W. Weitschat & W. Lourenço).

In 1995, a new specimen of scorpion from the Baltic amber was discovered in Hamburg, Germany. Upon examination of its visible characters, it was identified as a member of the family Buthidae (Fig. 2), belonging to a new genus and species, allied to the genus *Lychas* C. L. Koch, 1845 [28]. However, nothing could associate this specimen with the two species previously described by Holl [20] and Menge [21]. Subsequent studies revealed significant findings, leading to the discovery and description of over 10 specimens representing several new genera and species since 1996 [29, 30, 31, 32, 33, 34, 35]. These discoveries confirmed the relationship between this extinct fauna and elements of the extant buthid fauna found in both the Old and New Worlds. All scorpions found among the Baltic amber fauna belong to the family Buthidae and represent one or two generic lineages.

**Figure 3.**
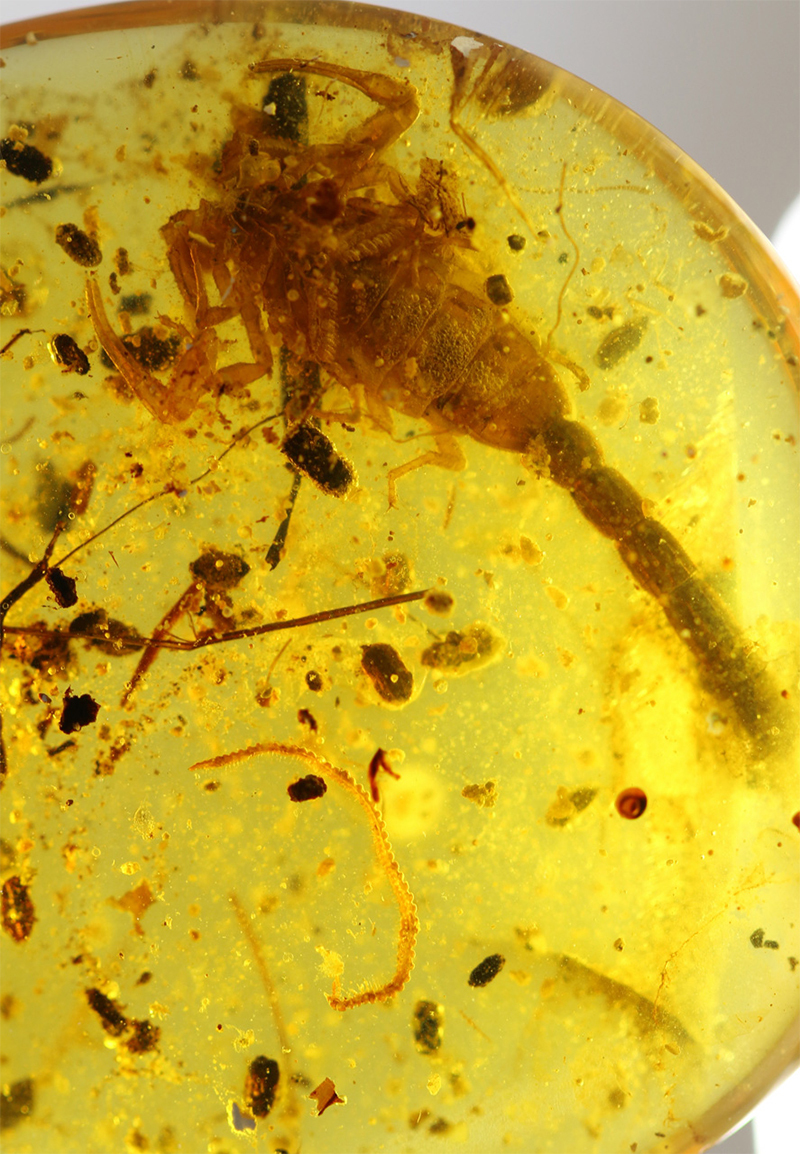
*Archaeoananteroides maderai*, holotype, ventral aspect (photo, J. Velten & W. Lourenço).

**Figure 4.**
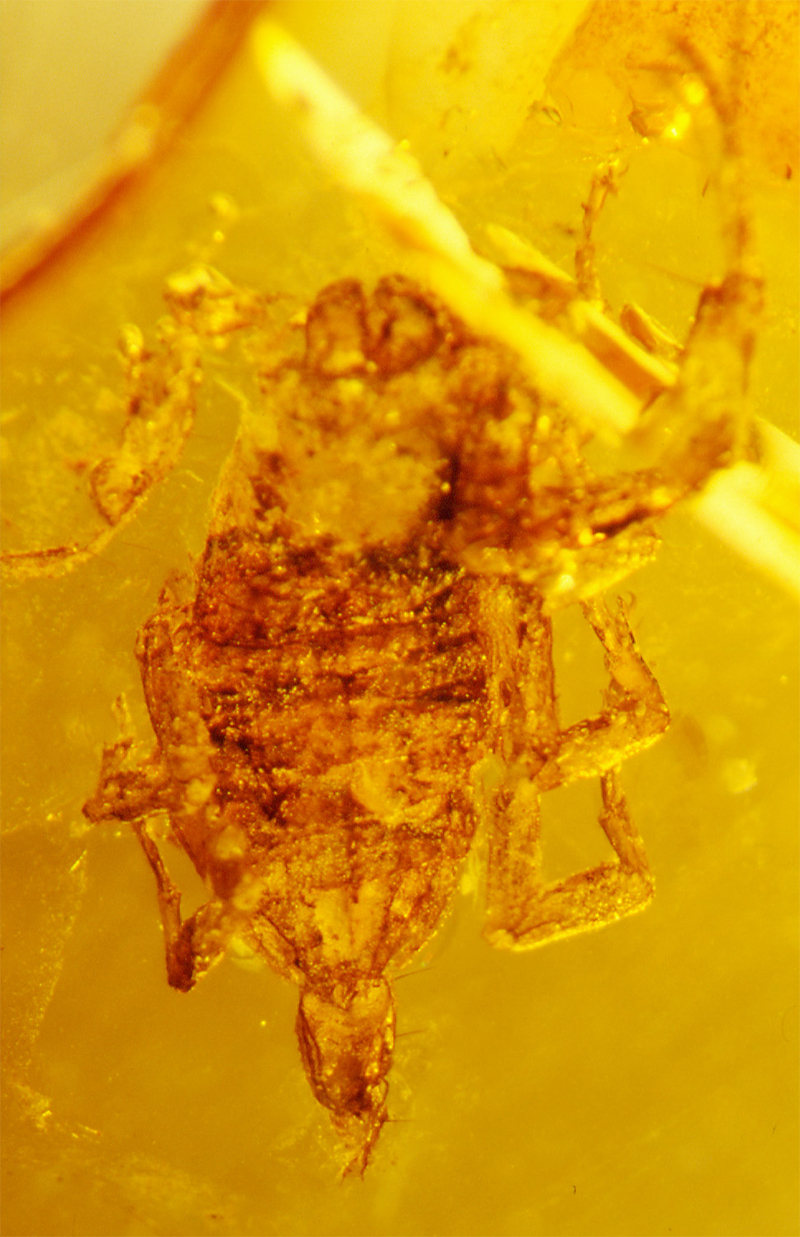
*Archaeobuthus estephani*, holotype, dorsal aspect (photo, W. Lourenço).

**Figure 5.**
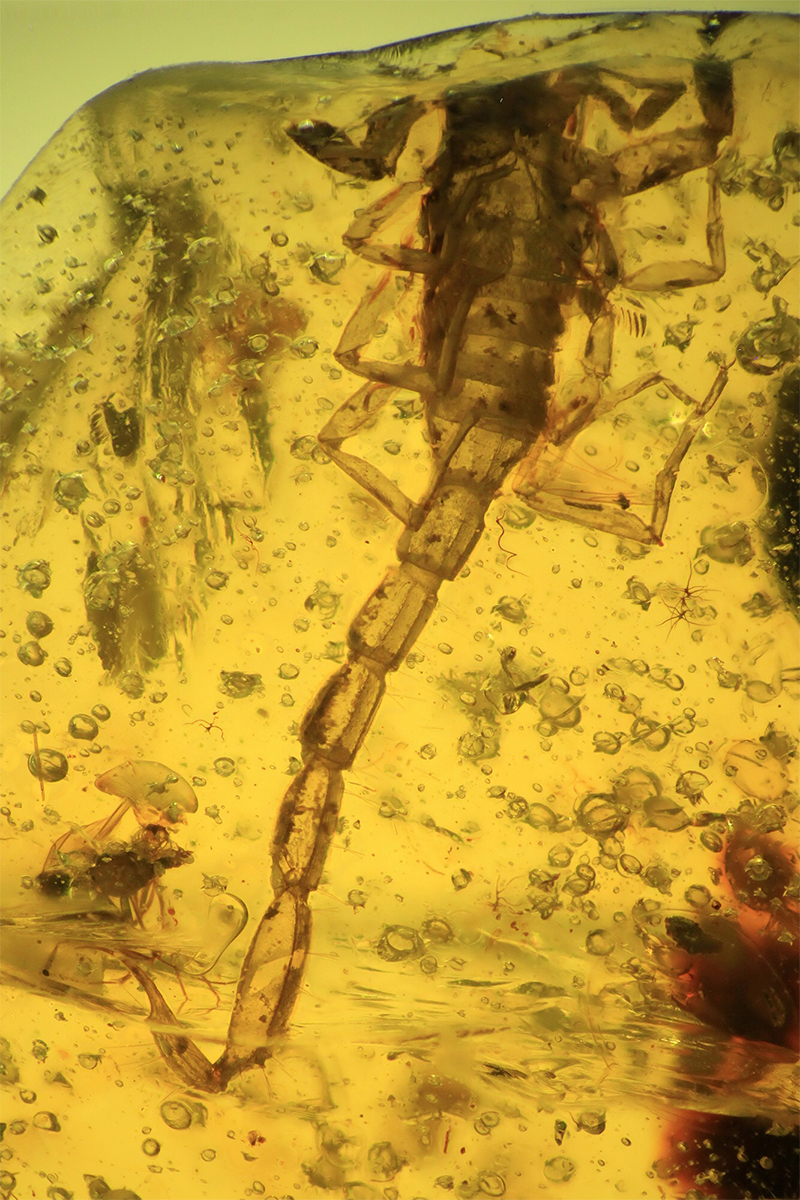
*Palaeoburmesebuthus knodeli*, holotype, dorsal aspect (photo H. Knodel).

**Figure 6.**
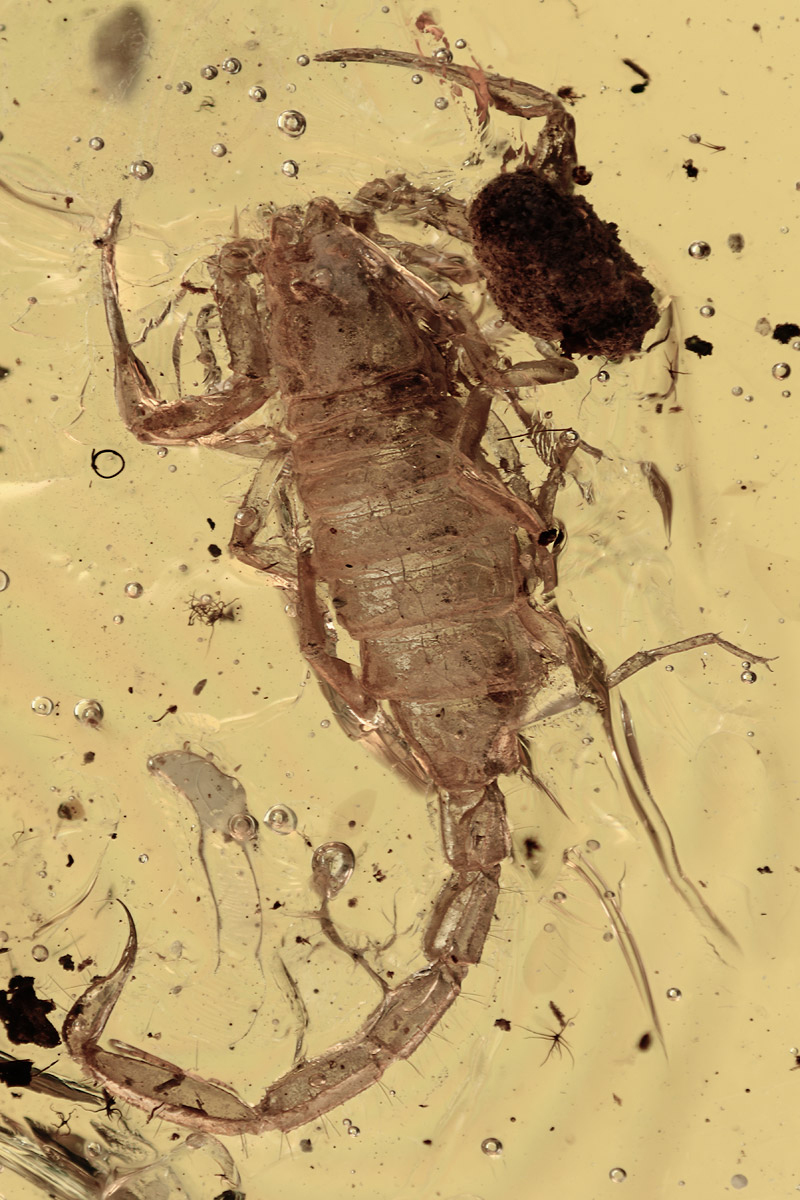
*Betaburmesebuthus bellus*, holotype, dorsal aspect (photo, C. Gröhn).

**Figure 7.**
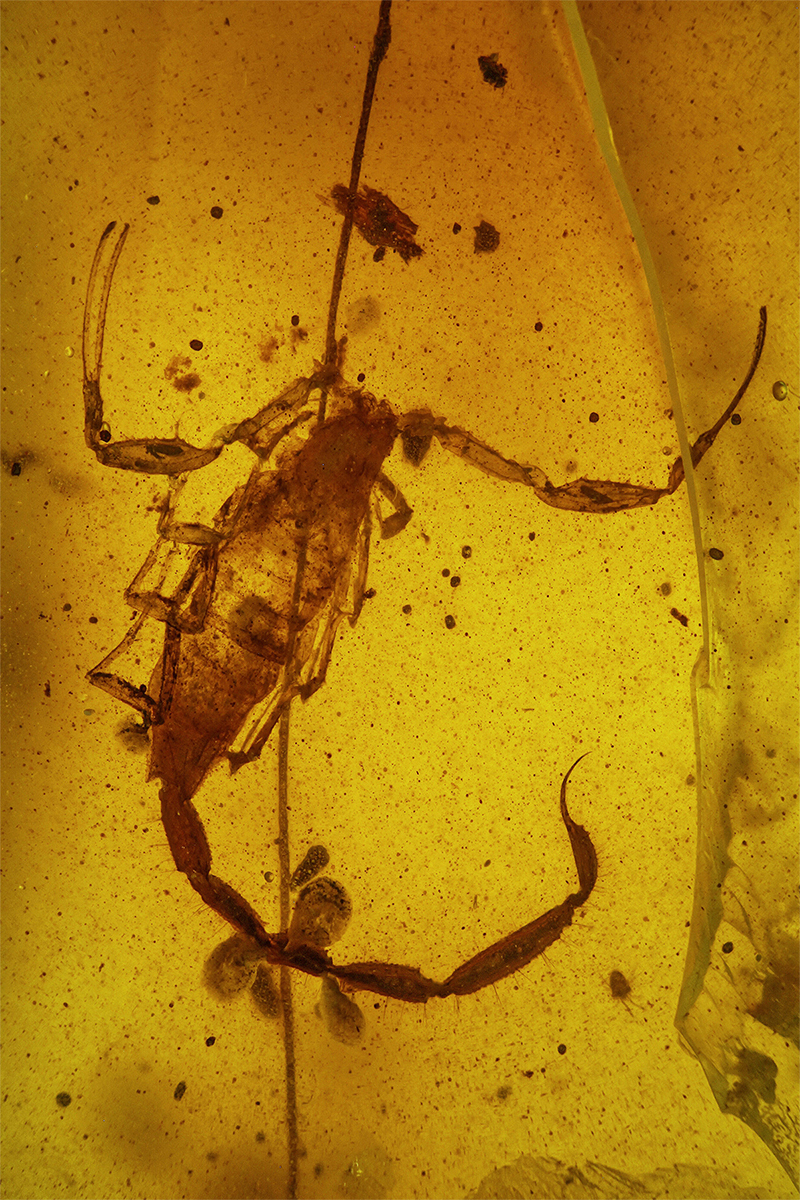
*Spinoburmesebuthus knodelorum*, holotype, dorsal aspect (photo, H. Knodel).

**Figure 8.**
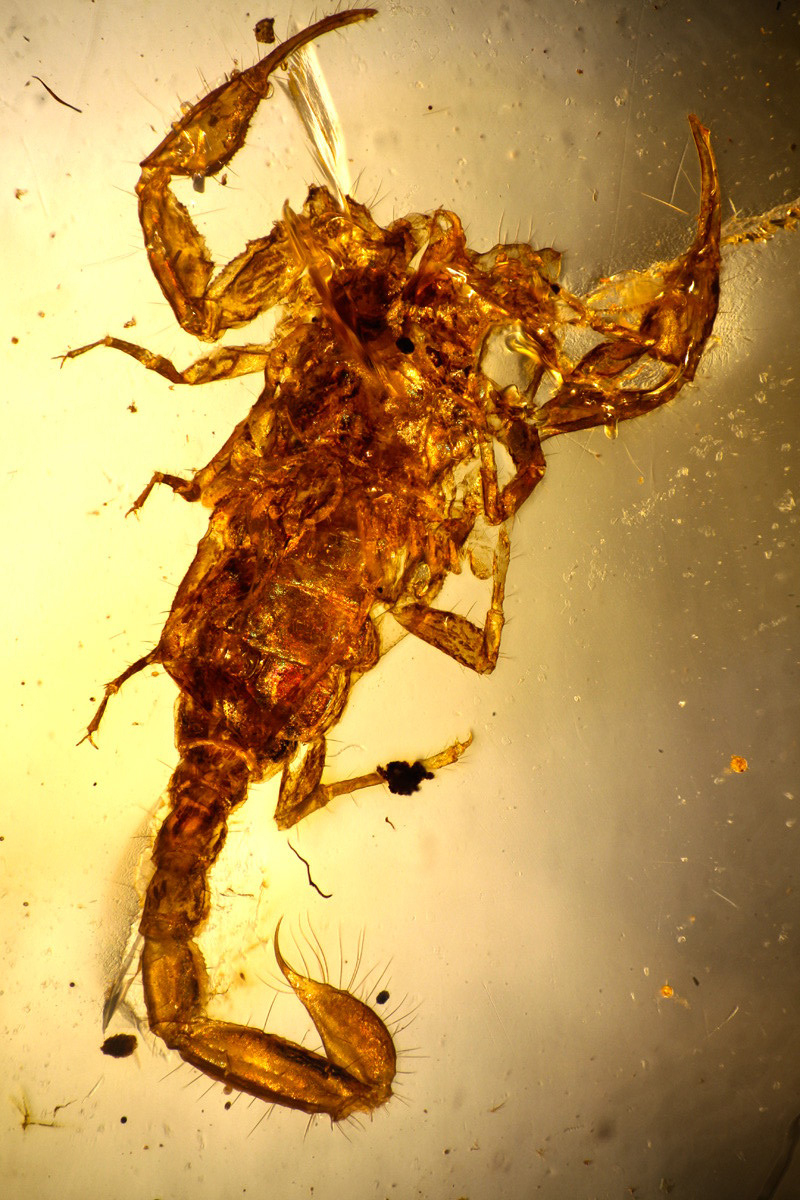
*Chaerilobuthus schwarzi*, holotype, dorsal aspect (photo, J. Velten & W. Lourenço).

**Figure 9.**
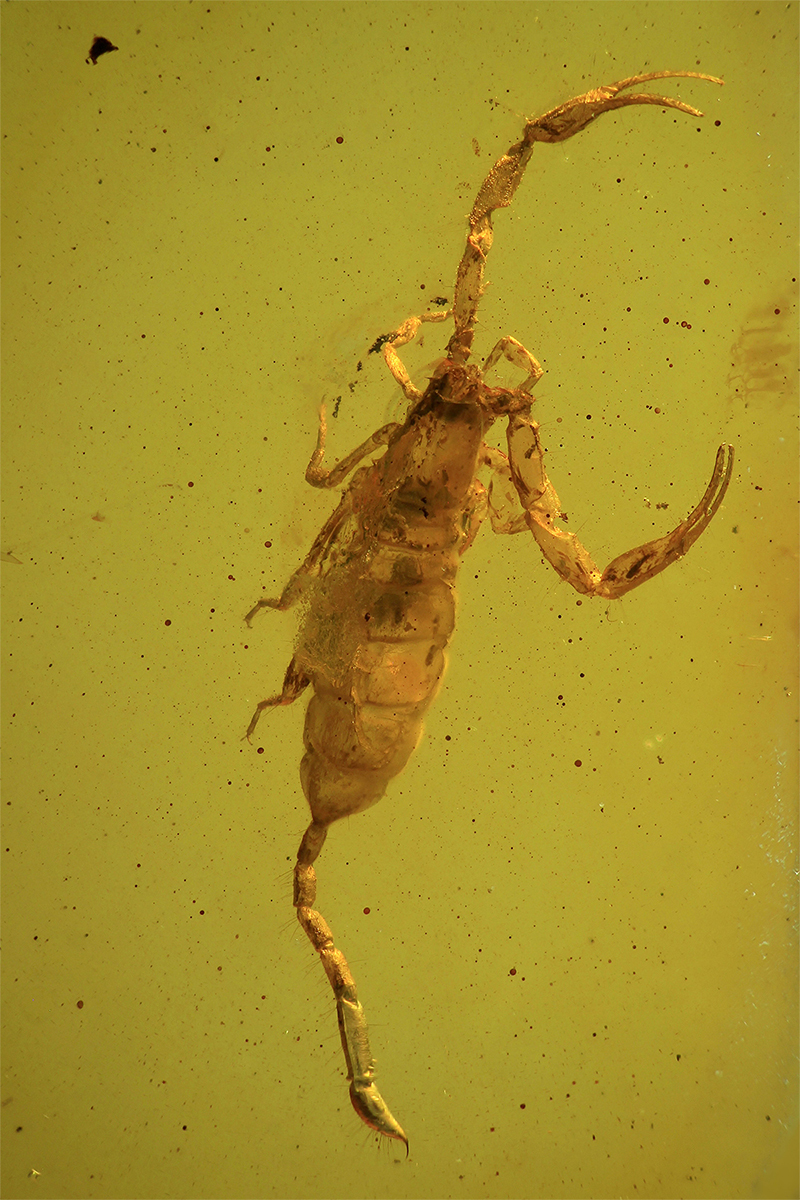
*Cretaceoushormiops knodeli*, holotype, dorsal aspect (photo, H. Knodel).

**Figure 10.**
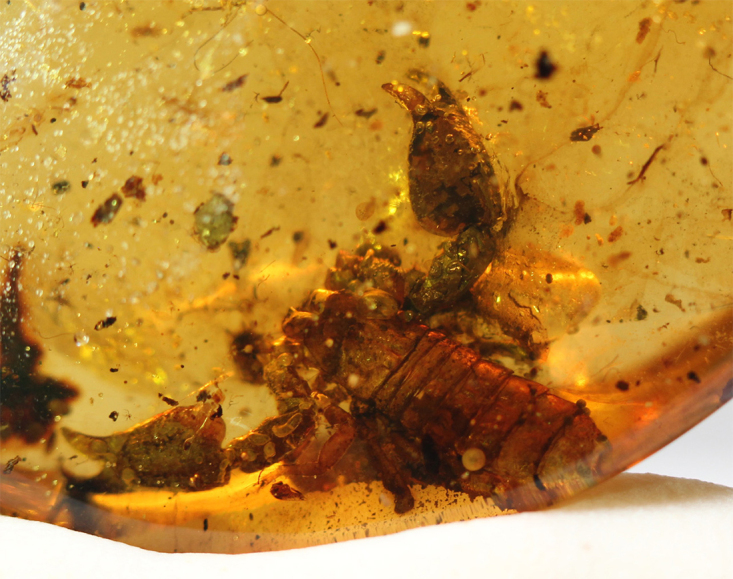
*Cretaceousopisthacanthus smeelei*, holotype, dorsal aspect (photo, J. Velten & W. Lourenço).

Even more significant were the discoveries and descriptions of scorpions found in Cretaceous amber, which began in 2001. These findings revealed several new families and subfamilies and a noticeable number of new genera and species. These fossil scorpions trapped in Cretaceous amber can be dated from 135 to 90 My BP. An important number of these elements can be associated with buthoids, and more precisely with buthids, such as two Burmite genera *Archaeoananteroides* Lourenço, 2016 (Fig. 3) and *Cretaceousbuthus* Lourenço, 2022, which have been accommodated in the family Buthidae C. L. Koch, 1837 [36,37]. Others buthoids were accommodated in their own families, such as *Archaeobuthus estephani* Lourenço, 2001 (Fig. 4), family Archaeobuthidae Lourenço, 2001[38], from Lebanon amber and the numerous species of the genera *Palaeoburmesebuthus* Lourenço, 2002, *Betaburmesebuthus* Lourenço, 2015 and *Spinoburmesebuthus* Lourenço, 2017 (Figs. 5-7), family Palaeoburmesebuthidae Lourenço, 2015 [39, 40, 41], all three equally from amber of Myanmar (Burmite). A remarkable number of non-buthoid elements have also been recorded and described. These comprise *Palaeoeuscorpius gallicus* Lourenço, 2003, family Palaeoeuscorpiidae Lourenço, 2003 from French amber [42] and several elements from Burmite, such as *Electrochaerilus buckleyi* Santiago-Blay, Fet, Soleglad & Anderson, 2004, family Chaerilidae Pocock, 1893 [43], and most significant, a noticeable number of species in genera *Chaerilobuthus* Lourenço & Beigel, 2011 (Fig. 8) [44] and *Chaeriloiurus* Lourenço, 2020 [45], family Chaerilobuthidae Lourenço & Beigel, 2011, *Palaeotrilineatus ellenbergeri* Lourenço, 2012, family Palaeotrilineatidae Lourenço, 2012 [46], *Archaeoscorpiops cretacicus* Lourenço, 2015 and *Burmesescorpiops groehni* Lourenço, 2016 [47, 48], in a new subfamily Archaeoscorpiopinae Lourenço, 2015, family Palaeoeuscorpiidae Lourenço, 2003 and *Sucinlourencous adrianae* Rossi, 2015, family Sucinlourencoidae Rossi, 2015 [49]. More recently, new non-buthoid elements have been described in the family Protoischnuridae Carvalho & Lourenço, 2001 [50], previously created to accommodate a sedimentary fossil from Brazilian Cretaceous, *Protoischnurus axelrodorum* Carvalho & Lourenço, 2001: *Cretaceoushormiops* Lourenço, 2018 with two species *Cretaceoushormiops knodeli* Lourenço, 2018 (Fig. 9) and *Cretaceoushormiops staxi* Lourenço, 2022 [51, 52] and *Cretaceousopisthacanthus* Lourenço, 2021 (Fig. 10) with one species *C. smeelei* Lourenço, 2021 [53]. In their totality, the non-buthoids discovered in Burmite may represent five distinct lineages. Including the buthoids, this number can be raised to six or seven, attesting therefore to the remarkable diversity present in the Cretaceous amber forests of Myanmar. Dated at almost 135 My BP, *Archaeobuthus estephani* remains the oldest known fossil scorpion ever discovered in amber [38].

## The origin of amber

The origins of fossilized resins or amber have been extensively discussed in numerous publications, such as the comprehensive study by Zherikhin and Ross [54]. Since I am not an expert on both the botanical and geological aspects of resin production and fossilization, I will provide a summary based on the very didactical article recently authored by Matuszewska [55].

Natural resins are the viscous secretions of plants, particularly seed-bearing woody plants. As products of trees, these can be secreted by both conifers (gymnosperms) and broadleaf trees (angiosperms). Even if the original *Araucaria* Juss., 1789 was already present in the Jurassic (in North Hemisphere), coniferous trees of the family Araucariaceae Henkel & W. Hochst., 1865 which created numerous fossil resin sites, have exclusively been described from Cretaceous. With one exception, Araucarias are currently absent from the Northern Hemisphere, while pine trees are prevalent.

During the Cretaceous period, a significant number of resins can be traced back to gymnosperm trees, specifically those belonging to the Araucariaceae family. This geological period also witnessed a rapid development of angiosperm plants (flowering plants), which coincided with the emergence of insects that interacted with them, including those that caused them harm. The Lower Cretaceous, in particular, played a crucial role in the coevolutionary process between insects and flowering plants.

Following the Cretaceous period, there was a significant increase in resin secretions during the Tertiary era, particularly in the Eocene, Oligocene, and Lower Miocene epochs. These resinous deposits are predominantly associated with Baltic amber or Succinite. The Eocene forests, which existed approximately 40-50 million years ago, covered the regions of present-day Scandinavia. These forests bore a striking resemblance to modern subtropical forests. The prevailing palaeoclimatic conditions during this period provided optimal conditions for the abundant growth of resin-producing trees. However, these conditions also facilitated the proliferation of harmful insects, which could attack the resinous trees and trigger defensive responses, such as the expulsion of resin.

Following the expulsion of the resin by the tree, the volatile fractions of the fresh resin evaporate, to act as a repellent. Subsequently, the resin’s viscosity increases and the non-volatile fractions solidify as its molecules get closer to each other and begin to undergo the process of condensation or polymerization and consequently, the resin hardens. The full process in nature may take long periods. Resins globally present a highly resistant chemical structure since the main purpose is to protect the tree. Nevertheless, as is any organic material, it will be subjected to destructive processes, except if it is covered with layers of water, soil, or rock. It seems that seawater environments may be favorable to the resin's survival.

**Figure 11.**
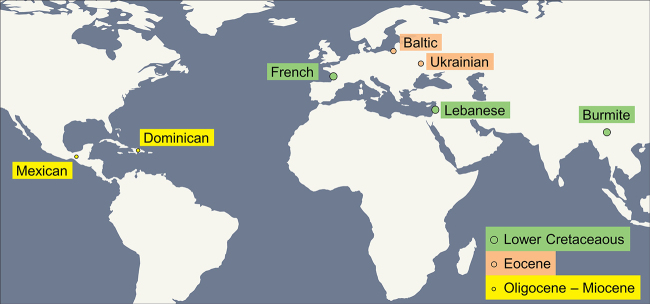
World map with indication of the sites where scorpions trapped in amber have been found (copywriter, L. Wilmé).

Certain similarities which characterize the Cretaceous resins (which contain the largest number of scorpion lineages) found in Asia, Europe, or North America (Fig. 11) are certainly related to the fact that these areas were located during the Cretaceous in the supercontinent-Laurasia (for more details refer to [55]).

## Comments on the approaches used in the study of amber fossils

**Figure 12.**
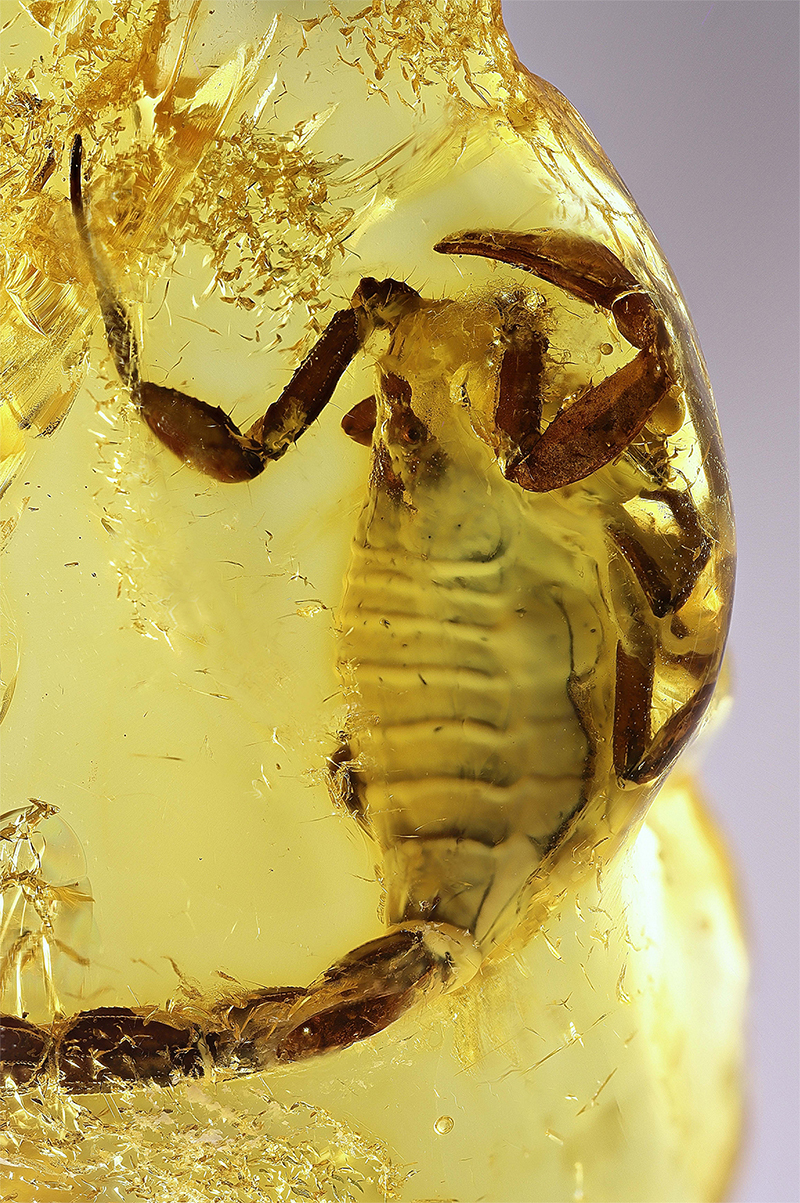
*Palaeolychas weitschati* from Baltic amber, covered by the milk-like substance (photo, J. Damzen).

The investigation of specimens trapped in amber follows a similar protocol to the one used in the study of recent specimens. Naturally, some limitations exist, such as the quality of the amber itself, the position of the specimen inside the resin, and the quality of its preservation. In many cases, many characters are not visible or are simply not observable at all. This is often the case for the trichobothrial pattern when bothria are extremely small and hairs are no longer present. For this reason, this very important character in the definition of scorpion lineages is often neglected by some authors. Besides, particular problems can be present; specimens in Baltic amber can be surrounded, at least in part, by a milk-like substance (Fig. 12). Burmite specimens often suffer different degrees of dissection within the resin that complicates a precise analysis of some characters.

The total number of scorpions described from Middle America or Baltic amber remains yet limited, and in most cases, the specimens used in their descriptions are complete or almost complete. In other cases, such as for Cretaceous French and Lebanon amber, specimens are not complete or can even be represented by a single fragment. However, due to their rarity, the definition of the new taxa generally still holds.

**Figure 13.**
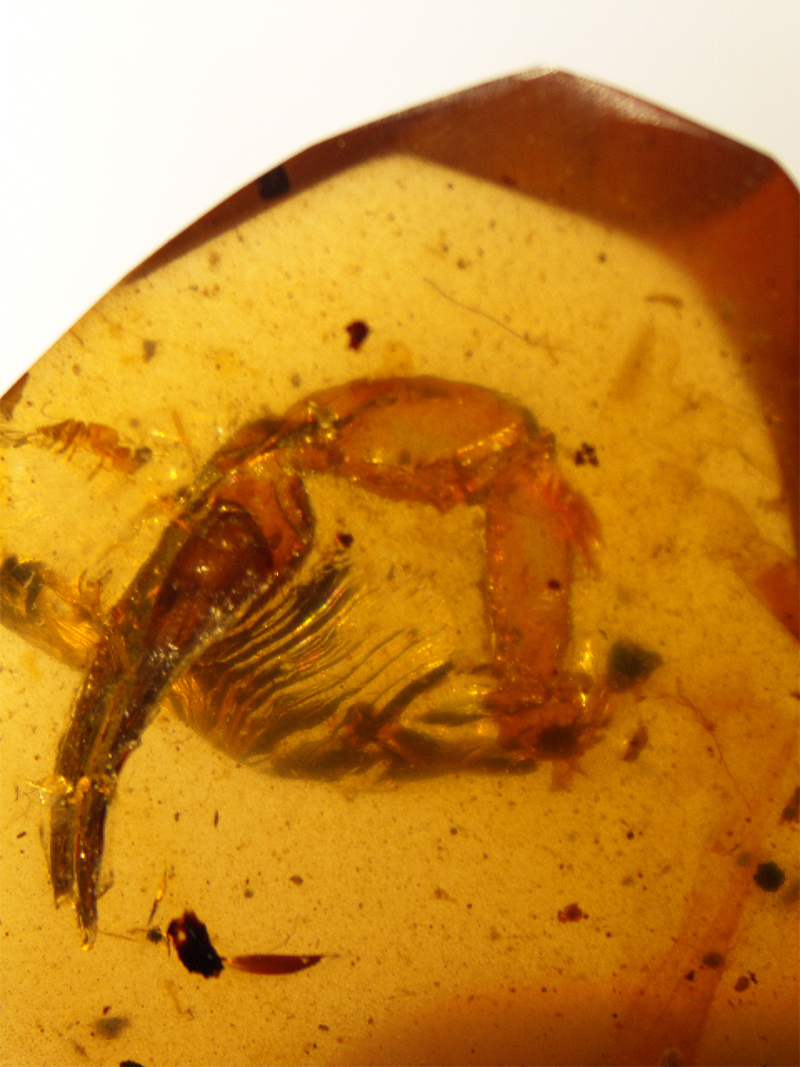
*Archaeoscorpiops cretacicus*, single pedipalp, which represents the holotype (photo, W. Wunderlich).

The situation concerning the studies of specimens found in Burmite can be rather variable. The first element ever described as *Palaeoburmesebuthus grimaldii* Lourenço, 2002 was largely incomplete, but due to its novelty, a new genus and species were proposed, even if placed in an *Incertae sedis* family [39]. Subsequently, other new taxa were based on incomplete specimens or even on fragments (Fig. 13), which nevertheless represented distinct lineages [47].

The quality of the Burmite specimens was discussed in several previous papers published in recent years, and comments were addressed about the remarkable pace observed in the studies of new scorpions found in Burmite [37, 53, 56, 57]. This pace of descriptions may pose challenges for future identifications, as many species found in Burmese amber exhibit similar morphologies. Therefore, as more taxa are discovered within a particular group, higher-quality specimens are needed for precise descriptions [51, 52, 53, 57, 58].

A crucial aspect of new descriptions is having a good understanding of all previously described fossils. Only this form of procedure can authorize new descriptions without the risk of misidentifications. However, it is challenging to fulfill this requirement as the majority of previously described specimens are typically held in private collections. Only a small percentage of the existent pieces are, when possible, correctly studied and classified, but it seems obvious that many if not most of them are never examined by any scorpion expert. Consequently, theoretical speculations or ‘recommendations’, as those recently proposed by Santiago-Blay et al. [12] may not serve the intended purpose and can seem futile or akin to pontification.

The taxonomical problem faced by the family Palaeoburmesebuthidae is a clear example of the challenges encountered in distinguishing and describing new species. In recent publications [12, 13, 14], new species were described, sometimes based on incomplete specimens [12], and accommodated in a particular genus in a ‘random decision’ without a final precision (see comments in the check-list below); this, as a consequence of the important similarity of the species. As a result, I have refrained from describing any new species in the genera *Palaeoburmesebuthus* and *Betaburmesebuthus* since 2018. Nevertheless, during these 5-6 years, I was able to examine more than 20 specimens belonging to this family. Instead of describing new species, I preferred, when possible, to better characterize some previously described ones, which in every case are known from a single specimen [37, 52]. Even for the other groups found in Burmite, only a small fraction, less than 25% of the specimens I examined, led to the description of new taxa [57].

It is important to clarify that the presence of co-authors in several of my previous publications, particularly those related to Burmite, is primarily due to the involvement of amber enthusiasts and collectors who actively search for specimens and maintain personal collections. Their participation is crucial for gaining access to the material necessary for research. However, it should be noted that the complete taxonomic responsibility for these publications lies solely with me. While the collaboration and assistance of these co-authors are valuable, I bear the ultimate responsibility for the taxonomic classifications and descriptions presented in the publications.

## Some comments on the taxonomy of scorpions found in different types of amber

### Tertiary amber from the Dominican Republic and Mexico

**Figure 14.**
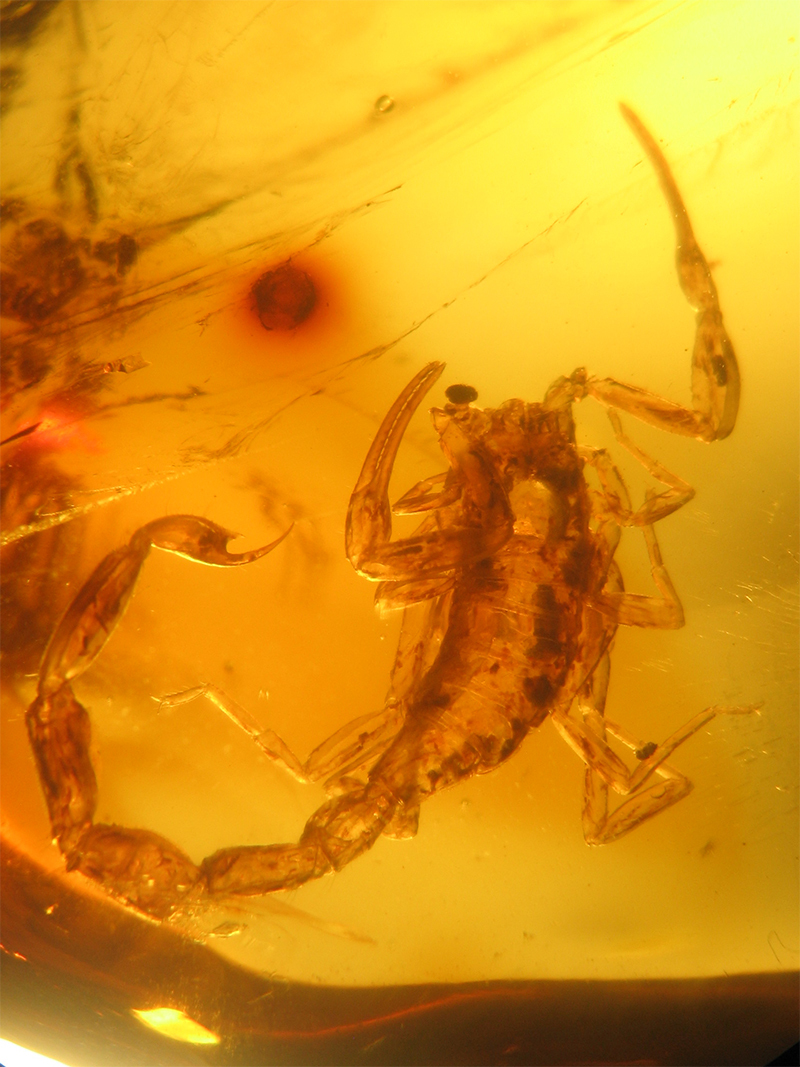
*Tityus hartkorni*, holotype, dorsal aspect (photo, J. Hartkorn).

**Figure 15.**
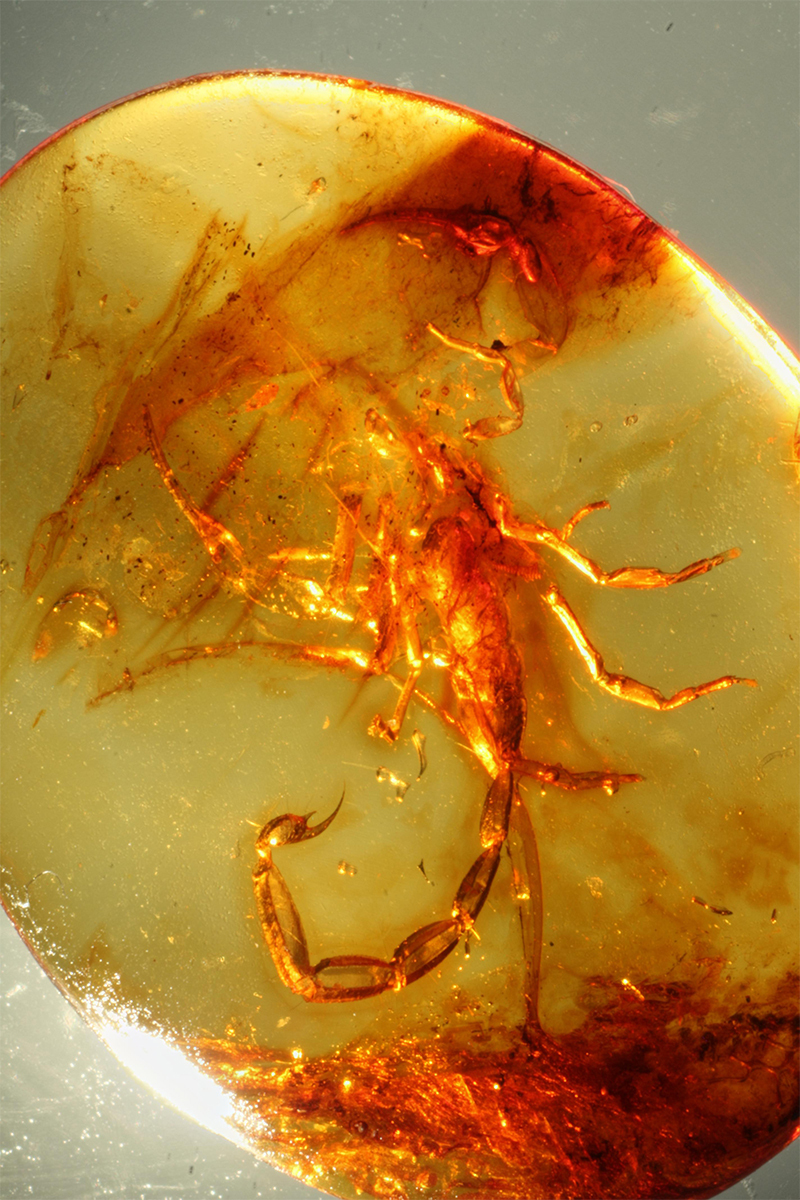
*Rhopalurus renelauerae*, holotype, dorsal aspect (photo, J. Velten & W. Lourenço).

This type of amber is rather recent, but the precise age remains often imprecise; one can estimate values ranging from 15 to 20 million years, basically Neogene/Miocene. Mainly because this amber is geologically recent, the scorpions trapped in it can be considered very close to elements of the extant fauna presently found in Tropical Americas. In their globality, they belong to the family Buthidae and up to now to the genera *Centruroides* Marx, 1890, *Tityus* C. L. Koch, 1836 and *Rhopalurus* Thorell, 1876. Some recently discovered examples are *Tityus* (*Brazilotityus*) *hartkorni* Lourenço, 2009, *Tityus azari* Lourenço, 2013, *Rhopalurus renelauerae* Lourenço, 2016, from Dominican Republic and *Tityus apozonalli* Riquelme, Villegas & Gonzalez, 2015 and *Centruroides knodeli* Lourenço, 2017 from Mexico (Figs. 14, 15).

### Tertiary Baltic Amber

Baltic amber is equally Tertiary but older than North and Central America's amber. Its age is estimated to be from the Palaeogene/Eocene, although some suggest that the current site where the amber is located may be redeposited, indicating a potentially older age. A reasonable estimation places its age between 55 to 60 My BP. All the scorpion specimens found in Baltic amber belong to the family Buthidae but are classified under different genera compared to those found in the extant global faunas. However, it is noteworthy that these genera show close relationships to the extant subfamily Ananterinae Pocock, 1900, which has a wide geographic distribution today.

The first discoveries date back to the early 19^th^ century, but only more recently some elements brought a more precise view of this Baltic amber fauna. Baltic amber was the first to provide fossil scorpions but these remain extremely rare when compared to Burmite scorpions. Since 1996, only 10 species have been described and accommodated in seven distinct genera. Six of these genera are associated with the extant Ananterinae while one genus *Palaeospinobuthus* Lourenço, Henderickx & Weistchat, 2005 appears to be closer to the Middle-East genus *Birulatus* Vachon, 1974 [59].

### Cretaceous amber

*Archaeobuthus estephani* Lourenço, 2001 (family Archaeobuthidae Lourenço, 2001) was the first scorpion described from Cretaceous amber and remains the oldest one known. This description was followed by that of *Palaeoburmesebuthus grimaldii* Lourenço, 2002, the first specimen found in Burmite; the new genus and species were however placed in an *Incertae sedis* family. Shortly after, a new rather incomplete specimen was found in French Cretaceous amber, but due to its unique characteristics, it led to the description of a new family, genus, and species: Palaeoeuscorpiidae Lourenço, 2003, *Palaeoeuscorpius* Lourenço, 2003 and *Palaeoeuscorpius gallicus* Lourenço, 2003.

Following these descriptions, no further scorpion specimens were found in Lebanese and French amber. However, the discoveries in Burmite have been increasingly abundant, resulting in the description of numerous taxa. Consequently, a huge amount of discoveries done in Burmite during the last 20 years led to the description of six families or subfamilies, 15 genera or subgenera, and 43 species (see check-list at the end of the article). Particularly noteworthy is the identification of several distinct lineages, numbering at least five to seven. However, further investigation is required to definitively determine the positions of at least two of these lineages.

The remarkable richness of Burmite amber is equally conducted to the pernicious attitude of several authors. The rising number of discoveries conducted certain authors, who certainly have poor access to specimens, to proceed in typical forms of downplay; this attitude is commonly used by authors, who in many cases rarely propose original results, or at most propose rather weak results surrounded by previous results of other authors, leading to a marked form of plagiarism.

This approach employed by these authors serves as a standard method for them to gain recognition at the expense of others’ discoveries. In very rare cases only, the originally described material is re-examined, but worse, in most cases the critics are only based on the original data of the authors they are precisely criticizing. When dealing with systematic studies on a given taxonomic group, it should be an obliged routine to refer to and consider the available material cited in previous publications. It is also noticeable to consider that since available fossil specimens are extremely rare, this paucity of information makes any re-evaluation more challenging; consequently, it remains unclear how much of a value it is to base re-evaluations on the same case specimens. Ironically, one can observe the emergence of new authors who emphasize the importance of their discoveries by discrediting previous work through shortcuts, neglecting detailed taxonomic treatments, which is paradoxical.

One notable example, which is becoming a classical one, is the controversial opinions of different authors concerning the presence of buthoid lineages during Mesozoic times. Polemic, more recently, also rose for chaeriloids.

After the clarification of the familial status of the genus *Palaeoburmesebuthus*, and consequently, of the genus *Betaburmesebuthus*, and their placement in the subfamily Palaeoburmesebuthinae, this last subfamily was temporarily accommodated in the family Archaeobuthidae Lourenço, 2001, both because of their association to the buthoid lineage, but in particular because of their similar geological horizon. Nevertheless, the subsequent study of several almost perfectly preserved specimens, clearly demonstrated their relationship to the buthoids [40, 56], in particular, based on their trichobothrial patterns which are almost identical to those of several extant buthoids. Based on these new characters, the subfamily Palaeoburmesebuthinae was raised to the family level as Palaeoburmesebuthidae and placed in the superfamily Buthoidea. Nevertheless, the exclusion of the family Archaeobuthidae from the buthoids has been proposed by several authors [60, 61], based however, mainly on theoretical speculation. It is well known that both higher classification of scorpions in general and classification of fossils, in particular, are controversial issues that have been largely debated within scorpion taxonomy in particular over the past 20 years.

In the precise case of *Archaeobuthus estephani* Lourenço 2001 (family Archaeobuthidae), all the available data are based only on a unique but incomplete specimen. The validity of the family Archaeobuthidae was not questioned by itself, but authors such as Baptista et al. [60], clearly rejected its association with the buthoids. Nevertheless, the available data we currently have for this unique Lebanon amber fossil is still insufficient to proceed with a revision of the position of this taxon. Therefore, a final decision should be based on further investigations when new specimens may become available [38].

### Bio-ecological comments

Defining the precise palaeoecological conditions of the original environments in which amber was formed may be a challenging task; in special in the cases where the amber has undergone reworking. This seems to be the case for both Baltic amber and Burmite [54]. It appears however that many amber samples are often found in river deltas and beach areas, suggesting a possible association with saltwater environments [55]. Also, the presence of some syninclusions such as Piddocks, (Bivalvia, Pholadidae) in Burmite pieces, can suggest that the Cretaceous amber-producing forests of Myanmar could be located near estuarine or freshwater environments [62].

Some biological particularities can also be summarized for all types of scorpions in amber or in particular for some groups exclusively found in Burmite:

*The majority of the specimens trapped in the resin are juveniles, suggesting that larger specimens or adults may have been capable of escaping the resin.

**Figure 16.**
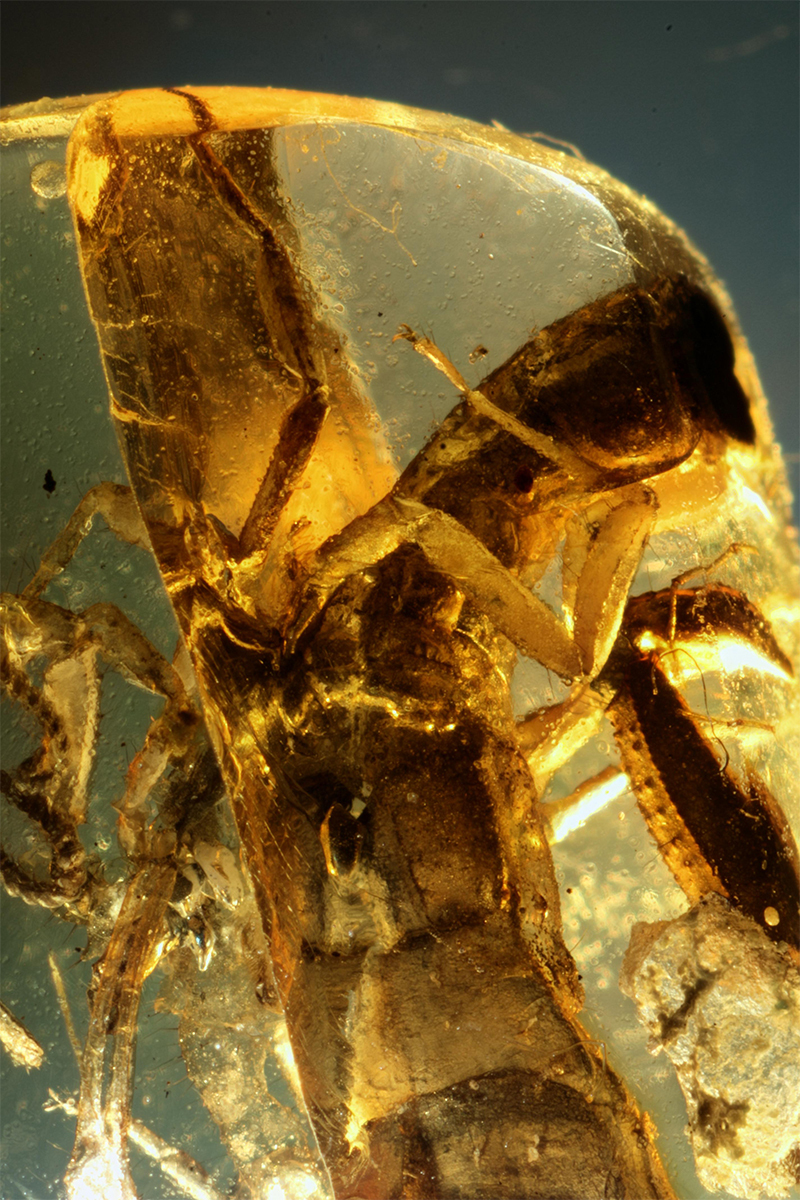
*Spinoburmesebuthus pohli*, holotype, in a molting process (photo, J. Velten & W. Lourenço).

*A significant portion of the studied specimens studied are represented by exuviae; probably abandoned under bark after the molting process. At least one example of a specimen trapped during the molting process (Fig. 16) is known for one species of *Spinoburmesebuthus* [41].

*The majority of the groups associated with the buthoids from all types of amber certainly correspond to bark scorpions, able to climb trees, as is the case for several extant buthid species. This is observed for the Middle-American lineages and those from Baltic amber. In the case of Burmite, the situation sounds more complex since several lineages correspond to non-buthoids. Since these elements are not supposed to climb trees, it can be suggested that these were trapped on the ground or very close to the base of the trees. This hypothesis was also suggested for the single known element described from Cretaceous French amber. This possibility would equally explain the larger size of some specimens, a priori, capable of escaping the trapping process.

*A final paradox, not explored, is the almost total absence of eyes in the species of the family Chaerilobuthidae. In this family, represented by the genus *Chaerilobuthus*, all the known species show a total absence of eyes, or in some cases very reduced eyes [44, 63]. This character is usually associated with cave-living species or in less frequent cases with species living in organic soil [64]. Given the lack of visual organs, it is unlikely that these scorpions would be capable of climbing trees. Therefore, it is highly probable that they were trapped by resin at ground level, indicating a different ecological niche compared to other scorpion lineages found in amber.

## Conclusions

At this point we return to the question addressed early in this contribution: How can a good knowledge of these fossils help in understanding present scorpionism problems? Or in other terms, what can fossils tell us about the evolution of the poisonous apparatus and eventually of venoms?

In previous publications [7, 8, 9] I drew attention to the evolutionary history of scorpions and the possible evolution of their venomous apparatus. It is widely accepted that scorpions originated as aquatic organisms. In their evolutionary history, they almost certainly evolved from the Eurypterida (‘water scorpions’) since both groups share several common morphological features. Marine and amphibious scorpions most certainly predominated during the Carboniferous (359-299 My BP) and some species certainly reached the Permian (299-251 My BP) and even the Triassic (251-200 My BP) periods [65, 66]. The first unequivocally terrestrial (air-breathing) scorpion most certainly appeared on land during the late Devonian (416-359 My BP) or early Carboniferous [67, 68].

These early scorpions, most of which were aquatic or amphibian quickly radiated into an impressive number of superfamilies and families. All these non-terrestrial fossil scorpions have been placed in one suborder Branchioscorpionina Kjellesvig-Waering, 1986. Fossil scorpions, accepted as terrestrial forms, are classified in a distinct suborder Neoscorpionina Thorell & Lindström, 1885 together with extant families. The suborder Branchioscorpionina includes 18 to 21 superfamilies and 41 to 47 families according to different authors [69, 70]. These numerous lineages are a clear indication of their early and great success. Moreover, because the fossil record is rather fragmentary, these more than 20 superfamilies likely represent only a fraction of the total number that existed [67, 69]). It is evident, however, that only a few, possibly only one of these lineages, survived and radiated into the present day. Naturally, all extant scorpions live now inland.

The significant number of fossil scorpion families accepted by strict paleontologists creates a divergence of opinion among neontologists. This divergence indicates a taxonomic problem, and the difficulties of this type are often the result of different approaches in the studies performed by paleontologists and neontologists. The former typically works from higher taxonomic categories downwards, while the latter work from lower categories upwards [69].

Another important question that is often raised concerns the age of extant scorpion lineages. Until recently, modern scorpion lineages were estimated to have been present since the very early Cenozoic [69]). This estimation was based on very few fossil records available for the Cenozoic and Mesozoic periods. Very recent discoveries for both the Cenozoic and Mesozoic periods based on both sedimentary and amber fossils attested that some extant lineages or at least proto-elements of these lineages are most certainly much older and were already present in the Lower Cretaceous [36, 37, 38, 40, 42, 44, 50, 51, 53].

Without any exception, all the extant scorpion species possess venom glands. The presence of a telson with an aculeus and, in some cases, possibly tegumentary glands are also evident in several fossil scorpions from the Palaeozoic, Mesozoic, and Cenozoic [50, 68, 69].

Tegumentary glands are common in many arthropods and these probably evolved from the secretion of basic enzymes to more and more elaborate toxins, achieving to become complex venom glands. Based on the assumption that venom glands in scorpions have originally a predatory and digestive role, it is possible to suggest a process of coevolution between the mechanical pattern of predation and the venomous function. This hypothesis serves as a suitable model for the elements of the buthoid lineage which generally have slender and/or weak pedipalps.

The exact evolution of the telson remains unclear. The structure was already present in Eurypterids and is yet common in several arthropod groups such as Xiphosura (horseshoe crabs). This posterior-most division of the body of an arthropod is not however considered as a true segment since it does not arises in the embryo from teloblast areas as do real segments. As for its possible original function in scorpions, the following path can be suggested. The telson probably played a major mechanical role in predation, with the aculeus acting as a ‘spearhead’. Several fossil scorpions of the buthoid lineage show quite long aculei [56, 58]) and this is also the case of a few extant genera of buthids such as the genus *Buthacus* Birula, 1908 or *Buthiscus* Birula, 1905 [71]. Over time, tegumentary glands evolved in the telson’s vesicle, nevertheless, their primitive role was only associated with the digestion of prey. In contrast, several non-buthid groups evolved with mechanical techniques of predation with the development of very strong and well-armed pedipalps. These groups do possess venom glands; however, the use of venom (toxins) for the capture of prey remains rather facultative. Such groups have been present since at least the Early Cretaceous [50, 51, 52, 53].

Naturally, this previous argumentation, although of some interest, does not explain why some groups of extant buthids do possess very active venoms, in particular on mammals, while others do not. Some hypotheses can be highlighted. Buthoids most certainly appear as the most complex group since they represent about 50% of all known scorpions and are the only group to be distributed in all the biogeographic regions of the earth. Some authors insist on the possible ‘monophyletic’ character of this group of scorpions, it seems however that this should not be the case. Buthoids which most certainly comprise a distinct number of families may be represented by four to five different evolutionary gradients [5]. The buthoid species possessing venoms formed by complex mixtures of highly specific toxins belong, in all cases, to genera such as *Androctonus* Ehrenberg, 1828, *Buthus* Leach, 1815, *Leiurus* Ehrenberg, 1828, *Centruroides* or *Tityus*, which can be placed in a high or even very high evolutionary level within the familial lineage. Most biochemical and ecological studies are concentrated on these groups because they are responsible for most scorpion incidents but also because represented by conspicuous populations. In contrast, very few or almost no studies have ever been performed with the most primitive lineages, both because these do not represent any threat to humans and because these scorpions are often rare, e. g. *Ananteris* Thorell, 1891 or *Birulatus*.

Some sedimentary fossils from the early Triassic such as the family Protobuthidae Lourenço & Gall, 2004 can already be classified among elements of the buthoid in a broad sense [68]. However, no precise connections can be done to precise extant generic groups for instance. More recent Cretaceous amber fossils can suggest some early links with extant lineages, and some well-defined families such as the Archaeobuthidae from the Cretaceous of Lebanon and Palaeoburmesebuthidae from Burmite can be assigned to the buthoid lineage. The links, however between the most common Cretaceous Burmite genera, *Palaeoburmesebuthus* Lourenço and *Betaburmesebuthus* Lourenço with extant genera remain vague. These two Burmite elements show very primitive characters which vanished in recent forms [56, 58]; nevertheless, in a few other isolated cases, elements from Cretaceous Burmite attested to be directly associated with the Buthidae family and extant elements. Examples include the genera *Archaeoananteroides* and *Cretaceousbuthus* which were associated with the family Buthidae [36, 37].

Although Cenozoic sedimentary fossils are extremely rare [72], several Baltic amber elements from this period have been studied. Earlier elements from this period can be dated from the Palaeocene to Eocene [73]. All studied scorpions from this period were classified in the family Buthidae, and with one single exception, were all assigned to the subfamily Ananterinae Pocock, 1900 [32, 34] which can be classified among the lower evolutionary buthoid gradients [5]. All the extant elements belonging to the Ananterinae are globally not noxious and although rare, present a wide range of distribution over different continents such as Africa, tropical America, and Asia. The present pattern of distribution of the Ananterinae suggests a panbiogeographic model and the group was most certainly dominant over all emerged lands in the early Cenozoic.

Many late Cenozoic elements are also known from Dominican and Mexican amber. The datation of this American amber is normally suggested as Oligocene-Miocene. The characteristic trait of the elements found in this late Cenozoic amber is that all, without exception can be accommodated within typical extant groups such as *Centruroides*, *Tityus,* and *Rhopalurus* [61, 74, 75] which can be classified as the most evolved according to the evolutionary gradients defined for the buthoids [5]. There are no fossil records available for other African noxious groups such as *Androctonus* and *Leiurus*, but the fossil chronology suggests the evolution of noxious species, probably from the middle of the Cenozoic period, and correlates well with the hypothesis suggesting that mammal-specific toxins would have evolved during aridification of the Palearctic region during the Tertiary period [76]. The evolution of these more evolved buthoid groups certainly took place in many regions of all emerged lands. Their presence and somewhat, more located areas of distribution can largely be attributed to more recent geological and palaeoclimatic vicissitudes which took place from the middle to the end of the Cenozoic epoch and even during the more recent Pleistocene period. These events align with the patterns observed in scorpion biogeography, specifically the millennial/Pleistocene and ecological biogeography [77].

## Supplementary material

The following online material is available for this article:

Addendum - Commented check-list of the known amber scorpion species.

## References

[B1] Lourenço WR, Cuellar O (1995). Scorpions, scorpionism, life history strategies, and parthenogenesis. J Venom Anim Toxins.

[B2] Lourenço WR (2008). Parthenogenesis in scorpions: some history - new data. J Venom Anim and Toxins incl Trop Dis.

[B3] Lourenço WR (2018). Scorpions and life-history strategies: from evolutionary dynamics toward the scorpionism problem. J Venom Anim Toxins incl Trop Dis.

[B4] Lourenço WR (2020). Why does the number of dangerous species of scorpions increase? The particular case of the genus Leiurus Ehrenberg (Buthidae) in Africa. J Venom Anim Toxins incl Trop Dis.

[B5] Lourenço WR (2001). The scorpion families and their geographical distribution. J Venom Anim Toxins.

[B6] Lourenço WR (2014). A historical approach to scorpion studies with special reference to the 20th and 21st centuries. J Venom Anim Toxins incl Trop Dis.

[B7] Lourenço WR (2015). What do we know about some of the most conspicuous scorpion species of the genus Tityus? A historical approach. J Venom Anim Toxins incl Trop Dis.

[B8] Lourenço WR (2016). Scorpion incidents, misidentification cases and possible implications on the interpretation of results. J Venom Anim Toxins incl Trop Dis.

[B9] Lourenço WR (2018). The evolution and distribution of noxious species of scorpions (Arachnida: Scorpiones). J Venom Anim Toxins incl Trop Dis.

[B10] Lourenço WR (2022). Back to Tityus serrulatus Lutz & Mello, 1922 (Scorpiones: Buthidae): new comments about an old species. J Venom Anim Toxins incl Trop Dis.

[B11] Lourenço WR, Cloudsley-Thompson JL, Cuellar O, Eickstedt VRD, Barraviera B, Knox MB (1996). The evolution of scorpionism in Brazil in recent years. J Venom Anim Toxins.

[B12] Santiago-Blay JA, Soleglad ME, Fet V, Craig PR, Smith LB (2022). Two new species of Cretaceous scorpions from Burmese amber (Arachnida: Scorpiones: Palaeoburmesebuthidae). Life.

[B13] Xuan Q, Cai C, Huang D (2022). A new Palaeoburmesebuthidae scorpion from mid-Cretaceous Burmese amber (Arachnida: Scorpiones: Buthoidea). Cretac Res.

[B14] Xuan Q, Cai C, Huang D (2023). Revision of palaeoburmesebuthid scorpions in mid-Cretaceous amber from northern Myanmar (Scorpiones: Buthoidea). Palaeoentomology.

[B15] Schawaller W (1979). Erstnachweis eines Skorpions in Dominikanischem Bernstein (Stuttgarter Bernsteinsammlung: Arachnida, Scorpionida). Stuttg Beitr Naturk.

[B16] Schawaller W (1982). Zwei weitere Skorpione in Dominikanischem Bernstein (Stuttgarter Bernsteinsammlung: Arachnida, Scorpionida). Stuttg Beitr Naturk.

[B17] Santiago-Blay JA, Poinar GO (1988). A fossil scorpion Tityus geratus new species (Scorpiones: Buthidae) from Dominican amber. Hist Biol.

[B18] Santiago-Blay JA, Poinar GO (1993). First scorpion (Buthidae: Centruroides) from Mexican amber (lower Miocene to upper Oligocene). J Arachnol.

[B19] Santiago-Blay JA, Schawaller W, Poinar GO (1990). A new specimen of Microtityus ambarensis (Scorpiones, Buthidae), fossil from Hispaniola: Evidence of taxonomic status and possible biogeographic implications. J Arachnol.

[B20] Holl F (1829). Handbuch der Petrefactenkunde. S, Dresden (Hilscher).

[B21] Menge A (1869). Lebenszeichen vorweltlicher, im Bernstein eingeschlossener Thiere. Schriften der naturwissenschaftlicher Gesellschaft in Danzig.

[B22] Larsson SG (1978). Baltic Amber - a Palaeobiological Study.

[B23] Werner F (1935). Klassen und Ordnungen des Tierreich. Dr. HG. Bronns: Arachnoidea. Akadem.

[B24] Petrunkevitch A, Moore RC (1955). Treatise on Invertebrate Paleontology. Part P. Arthropoda 2. Chelicerata with sections on Pycnogonida and Palaeoisopus.

[B25] Petrunkevitch A (1958). Amber spiders in European collections. Trans Connect Acad Arts Sci.

[B26] Kjellesvig-Waering EN (1986). A restudy of the fossil Scorpionida of the world. Palaeontogr Am.

[B27] Spahr U (1993). Ergänzungen und Berichtigungen zu R. Keilbachs bibliographie und liste der Bernsteinfossilen - Verschiedene Tiergruppen, ausgenommen Insecta und Araneae. Stuttg Beitr Naturk.

[B28] Lourenço WR, Weitschat W (1996). More than 120 years after its description, the enigmatic status of the genus of the Baltic amber scorpion ‘Tityus eogenus’ Menge, 1869 can finally be clarified. Mitteilungen aus dem Geologisch-Paläontologischen Inst Univ Hamburg.

[B29] Lourenço WR, Weitschat W (2000). New fossil scorpions from the Baltic amber - implications for Cenozoic biodiversity. Mitteilungen aus dem Geologisch-Paläontologischen. Inst Univ Hamburg.

[B30] Lourenço WR, Weitschat W (2001). Description of another fossil scorpion from Baltic amber, with considerations on the evolutionary levels of Cenozoic Buthoidea. Mitteilungen aus dem Geologisch-Paläontologischen. Inst Univ Hamburg.

[B31] Lourenço WR, Weitschat W (2005). A new genus and species of fossil scorpion from a different kind of Baltic amber (Scorpiones, Buthidae). Mitteilungen aus dem Geologisch-Paläontologischen. Inst Univ Hamburg.

[B32] Lourenço WR, Weitschat W (2009). A new species of Palaeoananteris Lourenço & Weitschat, 2001, fossil scorpion from Ukrainian amber (Scorpiones, Buthidae). Bol Soc Entomol Aragonesa.

[B33] Lourenço WR, Henderickx H, Weitschat W (2005). A new genus and species of fossil scorpion from Baltic amber (Scorpiones, Buthidae). Mitteilungen aus dem Geologisch-Paläontologischen Inst Univ Hamburg.

[B34] Lourenço WR (2012). Further considerations on scorpions found in Baltic amber, with a description of a new species (Scorpiones: Buthidae). Euscorpius.

[B35] Lourenço WR (2023). Baltic amber scorpions; a new noticeable specimen is now deposited in the Museum of Gdansk (Muzeum Gdanska), Poland. Amb Mag.

[B36] Lourenço WR, Velten J (2016). A new genus and species of fossil scorpion from Burmese Cretaceous amber (Scorpiones: Buthoidea: Buthidae). Riv Aracnol Italiana.

[B37] Lourenço WR, Velten J (2023). Confirmation of the validity of the genus Cretaceousbuthus Lourenço, 2022 and description of a new species from Burmite (Scorpiones: Buthoidea: Buthidae). Faunitaxys.

[B38] Lourenço WR (2001). A remarkable scorpion fossil from Lebanon amber. Implications for the phylogeny of Buthoidea. C R Acad Sci.

[B39] Lourenço WR (2002). The first scorpion fossil from the Cretaceous amber of Myanmar (Burma). New implications for the phylogeny of Buthoidea. C R Palevol.

[B40] Lourenço WR, Beigel A (2015). A new genus and species of Palaeoburmesebuthinae Lourenço, 2014 (Scorpiones: Archaeobuthidae:) from Cretaceous amber of Myanmar. Beitr Araneol.

[B41] Lourenço WR, Velten J (2017). One more new genus and species of fossil scorpion from Burmese Cretaceous amber belonging to the family Palaeoburmesebuthidae (Scorpiones). Riv Aracnol Italiana.

[B42] Lourenço WR (2003). The first scorpion fossil from the Cretaceous amber of France. New implications for the phylogeny of Chactoidea. C R Palevol.

[B43] Santiago-Blay JA, Fet V, Soleglad ME, Anderson SR (2004). A new genus and subfamily of scorpions from Lower Cretaceous Burmese amber (Scorpiones: Chaerilidae). Rev Ibérica Aracnol.

[B44] Lourenço WR, Beigel A (2011). A new scorpion fossil from the Cretaceous amber of Myanmar (Burma). New phylogenetic implications. C R Palevol.

[B45] Lourenço WR, Velten J (2020). A new contribution to the knowledge of Cretaceous Burmese amber scorpions with the descriptions of two new genera and two new species (Scorpiones: Chaerilobuthidae: Palaeoeuscorpiidae: Archaeoscorpiopinae). Riv Aracnol Italiana.

[B46] Lourenço WR (2012). About the scorpion fossils from the Cretaceous amber of Myanmar (Burma) with the descriptions of a new family, genus and species. Acta Biol Paranaense.

[B47] Lourenço WR (2015). A new subfamily, genus and species of fossil scorpions from Cretaceous Burmese amber (Scorpiones: Palaeoeuscorpiidae). Beitr Araneol.

[B48] Lourenço WR (2016). A new genus and three new species of scorpions from Cretaceous Burmese amber (Scorpiones: Chaerilobuthidae: Palaeoeuscorpiidae). Arthropoda Sel.

[B49] Rossi A (2015). A new family, genus and species of scorpion from Burmite of Myanmar (Scorpiones: Sucinlourencoidae). Riv Aracnol Italiana.

[B50] Carvalho MGP, Lourenço WR (2001). A new family of fossil scorpions from the Early Cretaceous of Brazil. C R Acad Sci.

[B51] Lourenço WR (2018). A new remarkable scorpion genus and species from Cretaceous Burmese amber (Scorpiones: Protoischnuridae). Riv Aracnol Italiana.

[B52] Lourenço WR, Velten J (2022). A second new species for the genus Cretaceoushormiops Lourenço, 2018 from Cretaceous Burmite (Scorpiones: Protoischnuridae). Faunitaxys.

[B53] Lourenço WR, Velten J (2021). One more new genus and species of scorpion from Early Cretaceous Burmese amber (Scorpiones: Protoischnuridae). Faunitaxyx.

[B54] Zherikhin VV, Ross AJ (2000). A review of the history, geology and age of Burmese amber (Burmite). Bull Nat Hist Mus.

[B55] Matuszewska A (2023). Origin of fossil resins. Amber Mag.

[B56] Lourenço WR (2016). A preliminary synopsis on amber scorpions with special reference to Burmite species: an extraordinary development of our knowledge in only 20 years. Zookeys.

[B57] Lourenço WR, Velten J (2022). Further insights on Cretaceous Burmite scorpions with the descriptions of a new genus and species (Scorpiones: Buthoidea; Buthidae). Faunitaxys.

[B58] Lourenço WR (2021). Further comments on the elements of the family Palaeoburmesebuthidae Lourenço, 2015 with description of a new species of Spinoburmesebuthus Lourenço, 2017 from Early Cretaceous Burmite amber (Scorpiones). Faunitaxyx.

[B59] Lourenço WR, Al-Saraireh M, Afifeh BA, Baker MA, Katbeh AB, Amr Z (2021). New insights on the taxonomy of the genus Birulatus Vachon, 1974 (Scorpiones: Buthidae), and description of a new remarkable species from Jordan. Bull Soc Entomol France.

[B60] Baptista CJ, Santiago-Blay JA, Soleglad ME, Fet V (2006). The Cretaceous scorpion genus, Archaeobuthus, revisited (Scorpiones: Archaeobuthidae). Euscorpius.

[B61] Riquelme FG, Villegas-Guzmán E, González-Santillán V, Córdova-Tabares OF, Francke D, Piedra-Jiménez E, Estrada-Ruiz B (2015). New fossil scorpion from the Chiapas amber Lagerstätte. PloS One.

[B62] Bolotov IN, Aksenova OV, Vikhrev IV, Konoplev ES (2021). New fossil Piddock (Bivalvia: Pholadidae) may indicate estuarine to freshwater environments near Cretaceous amber-producing forests in Myanmar. Nature.

[B63] Lourenço WR (2015). Clarification of the familial status of the genus Palaeoburmesebuthus Lourenço, 2002 from Cretaceous Burmese amber (Scorpiones: Archaeobuthidae: Palaeoburmesebuthinae). Beitr Araneol.

[B64] Lourenço WR (2009). Eyeless forest litter scorpions; a new species from the island of Halmahera (Moluccas), Indonesia (Scorpiones, Chaerilidae). Bol Soc Entomol Aragonesa.

[B65] Briggs DEG (1987). Scorpions take to the water. Nature.

[B66] Shear WA, Kukalová-Peck J (1990). The ecology of Paleozoic terrestrial arthropods: the fossil evidence. Canadian J Zool.

[B67] Jeran AJ, Brownell P, Polis GA (2001). Scorpion Biology and Research.

[B68] Lourenço WR, Gall JC (2004). Fossil scorpions from the Buntsandstein (Early Triassic) of France. C R Palevol.

[B69] Sissom WD, Polis GA (1990). The Biology of Scorpions.

[B70] Fet V, Fet V, Sissom WD, Lowe G, Braunwalder ME (2000). Catalog of the Scorpions of the World (1758-1998).

[B71] Lourenço WR, Kourim ML, Sadine SE (2017). Scorpions from the region of Tamanrasset, Algeria. Part I. A new species of Buthacus Birula, 1908 (Scorpiones: Buthidae). Riv Aracnol Italiana.

[B72] Kühl G, Lourenço WR (2017). A new genus and species of fossil scorpion (?Euscorpiidae) from the Early-Middle Eocene of Pesciara (Bolca, Italy).

[B73] Weitschat W, Wichard W (1998). Atlas der Pflanzen und Tiere im Baltischen Bernstein.

[B74] Lourenço WR (2017). A new species of Centruroides Marx, 1890 from Chiapas amber, Mexico (Scorpiones: Buthidae). Rev Ibérica Aracnol.

[B75] Lourenço WR, Velten J (2016). A new species of Rhopalurus Thorell, 1876 from Dominican amber (Scorpiones, Buthidae). Rev Ibérica Aracnol.

[B76] Fet VB, Gantenbein AV, Gromov G, Lourenço WR (2003). The first molecular phylogeny of Buthidae (Scorpiones). Euscorpius.

[B77] Lourenço WR (1996). The biogeography of scorpions. Rev Suisse Zool.

[B78] Xuan Q, Cai C, Huang D (2023). Immature chaerilid scorpions from mid-Cretaceous amber of northern Myanmar (Arachnida: Scorpiones: Chaeriloidea). Cretac Res.

[B79] Menon F (2007). Higher systematics of scorpions from the Crato Formation, Lower Cretaceous of Brazil. Palaeontology.

