# Supplementary material for: Scorpions trapped in amber: a remarkable window on their evolution over time from the Mesozoic period to present days
Source: J Venom Anim Toxins Incl Trop Dis. 2023 Aug 14;29:e20230040. doi: 10.1590/1678-9199-JVATITD-2023-0040 (PMC10430024; doi:10.1590/1678-9199-JVATITD-2023-0040)
Supplement: Addendum - [file 1678-9199-jvatitd-29-e20230040-s1.pdf]

Journal of Venomous Animals and Toxins including Tropical Diseases
https://doi.org/10.1590/1678-9199-JVATITD-2023-0040

Supplementary Material to "Scorpions trapped in amber: a remarkable window on their evolution over time from the Mesozoic period to present days"

ADDENDUM

Commented check-list of the known amber scorpion species

Middle America amber

Family **Buthidae** C. L. Koch, 1837

Genus ***Centruroides*** Marx, 1890

Centruroides beynai Schawaller, 1979 – Dominicain Republic

Centruroides knodeli Lourenço, 2017 – Mexico

Genus ***Microtityus*** Kjellesvig-Waering, 1966

Microtityus ambarensis (Schawaller, 1982); previously under *Tityus* - DR

Genus ***Rhopalurus*** Thorell, 1876

Rhopalurus renelauerae Lourenço, 2016 - DR

Genus ***Tityus*** C. L. Koch, 1836

Tityus geratus Santiago-Blay & Poinar, 1988 - DR

Tityus hartkorni Lourenço, 2009 - DR

Tityus azari Lourenço, 2013 - DR

Tityus knodeli Lourenço, 2014 - Mexico

Tityus apozonalli Riquelme, Villegas & Gonzalez, 2015 - Mexico

Tertiary Baltic Amber

Family: *Incertae sedis*

Scorpio schweiggeri Holl, 1829

Note: Probably a buthid.

Family **Buthidae** C. L. Koch, 1837

Genus *Tityus* C. L. Koch, 1836

Tityus eogenus Menge, 1869

Genus ***Palaeolychas*** Lourenço & Weitschat, 1996

Palaeolychas balticus Lourenço & Weitschat, 1996

Palaeolychas weitschati Lourenço, 2012

Genus ***Palaeotityobuthus*** Lourenço & Weitschat, 2000

Palaeotityobuthus longiaculeus Lourenço & Weitschat, 2000

Genus ***Palaeoprotobuthus*** Lourenço & Weitschat, 2000

Palaeoprotobuthus pusillus Lourenço & Weitschat, 2000

Genus ***Palaeoakentrobuthus*** Lourenço & Weitschat, 2000

Palaeoakentrobuthus knodeli Lourenço & Weitschat, 2000

Genus ***Palaeoananteris*** Lourenço & Weitschat, 2001

Palaeoananteris ribnitiodamgartensis Lourenço & Weitschat, 2001

Palaeoananteris wunderlichi Lourenço, 2004

Palaeoananteris ukrainensis Lourenço & Weitschat, 2009

Genus ***Palaeoisometrus*** Lourenço & Weitschat, 2005

Palaeoisometrus elegans Lourenço & Weitschat, 2005

Genus ***Palaeospinobuthus*** Lourenço, Henderickx & Weitschat, 2005

Palaeospinobuthus cenozoicus Lourenço, Henderickx & Weitschat, 2005

Lower Cretaceous amber from Lebanon

Family **Archaeobuthidae** Lourenço, 2001

Genus ***Archaeobuthus*** Lourenço, 2001

Archaeobuthus estephani Lourenço, 2001

Lower Cretaceous amber from France

Family **Palaeoeuscorpiidae** Lourenço, 2003

Genus ***Palaeoeuscorpius*** Lourenço, 2003

Palaeoeuscorpius gallicus Lourenço, 2003

Cretaceous Burmite

Family **Buthidae** C. L. Koch, 1837

Genus ***Archaeoananteroides*** Lourenço, 2016

Archaeoananteroides maderai Lourenço, 2016

Genus ***Cretaceousbuthus*** Lourenço, 2022

Cretaceousbuthus fraaijeorum Lourenço, 2022

Cretaceousbuthus petersi Lourenço, 2023

Family **Palaeoburmesebuthidae** Lourenço 2015

Genus ***Palaeoburmesebuthus*** Lourenço, 2002

Palaeoburmesebuthus grimaldii Lourenço, 2002

Palaeoburmesebuthus ohlhoffi Lourenço, 2015

Palaeoburmesebuthus longipalpis Lourenço & Rossi, 2017

Palaeoburmesebuthus knodeli Lourenço, 2018

Palaeoburmesebuthus smithi Santiago-Blay, Soleglad, Craig & Fet, 2022

Palaeoburmesebuthus andrewrossi Santiago-Blay, Soleglad, Fet, Craig & Smith, 2022

Note: These two species were placed in the genus *Palaeoburmesebuthus* (by default); they could equally belong to the genus *Betaburmesebuthus*.

Genus ***Betaburmesebuthus*** Lourenço, 2015

Betaburmesebuthus kobberti Lourenço, 2015

Betaburmesebuthus muelleri Lourenço, 2015

Betaburmesebuthus bidentatus Lourenço, 2015

Betaburmesebuthus fleissneri Lourenço, 2016

Betaburmesebuthus bellus Lourenço, 2016

Betaburmesebuthus larafleissnerae Lourenço, 2016

Betaburmesebuthus joergi Lourenço & Rossi, 2017

Betaburmesebuthus spinipedis Xuan, Cai & Huang, 2022

Note: As already suggested [57], this species most certainly belongs to the genus *Spinoburmesebuthus*.

Betaburmesebuthus villosus Xuan, Cai & Huang 2023

Note: A possible synonym of *Palaeoburmesebuthus smithi* Santiago-Blay, Soleglad, Craig & Fet, 2022.

Betaburmesebuthus fuscus Xuan, Cai & Huang 2023

Note: A possible synonym of one of the previously described species; probably *B. bellus*; the dark coloration is certainly an artifact due to the oxidation of resin.

Genus ***Spinoburmesebuthus*** Lourenço, 2017

Spinoburmesebuthus pohli Lourenço, 2017

Spinoburmesebuthus knodelorum Lourenço, 2021

Note: Xuan, Cai & Huang [14], suggested, based on very poor evidence, that *Spinoburmesebuthus* should be considered as a synonym of *Betaburmesebuthus*. This decision was certainly taken under the influence of some neontologists who know nothing about amber fossils. On account of this point, the genus *Spinoburmesebuthus* was revalidated in a recent re-analysis [37].

Family **Chaerilidae** Pocock, 1893

Subfamily **Electrochaerilinae** Santiago-Blay, Fet, Soleglad & Anderson, 2004

Genus ***Electrochaerilus*** Santiago-Blay, Fet, Soleglad & Anderson, 2004

Electrochaerilus buckleyi Santiago-Blay, Fet, Soleglad & Anderson, 2004

Note: Xuan, Cai & Huang in press [78], propose a *Chaerilus* sp. which could, in my opinion, be described as a new species. They also suggested that Electrochaerilinae and *Electrochaerilus* are considered dubious, without any justification. It seems reasonable that at least the genus *Electrochaerilus* should be maintained as valid.

Family **Chaerilobuthidae** Lourenço & Beigel, 2011

Genus ***Chaerilobuthus*** Lourenço & Beigel, 2011

Chaerilobuthus complexus Lourenço & Beigel, 2011

Chaerilobuthus longiaculeus Lourenço, 2013

Chaerilobuthus birmanicus Lourenço 2015

Chaerilobuthus bruckschi Lourenço, 2015

Chaerilobuthus schwarzi Lourenço, 2015

Chaerilobuthus enigmaticus Lourenço, 2015

Chaerilobuthus gigantosternum Lourenço, 2016

Chaerilobuthus serratus Lourenço, 2016

Chaerilobuthus knodelorum Lourenço, 2018

Chaerilobuthus hansgeorgmuelleri Lourenço, 2019

Chaerilobuthus meggeri Lourenço, 2021

Chaerilobuthus brandti Lourenço, 2022

Genus ***Chaeriloiurus*** Lourenço, 2020

Chaeriloiurus brigittemuellerae Lourenço, 2020

Family **Palaeotrilineatidae** Lourenço, 2012

Genus ***Palaeotrilineatus*** Lourenço, 2012

Palaeotrilineatus ellenbergeri Lourenço, 2012

Family **Palaeoeuscorpiidae** Lourenço, 2003

Subfamily **Archaeoscorpiopinae** Lourenço, 2015

Genus ***Archaeoscorpiops*** Lourenço, 2015

Archaeoscorpiops cretacicus Lourenço, 2015

Genus ***Burmesescorpiops*** Lourenço, 2016

Burmesescorpiops groehni Lourenço, 2016

Genus ***Chaeriloscorpiops*** Lourenço, 2020

Chaeriloscorpiops bautschi Lourenço, 2020

Family **Sucinlourencoidae** Rossi, 2015

Genus ***Sucinlourencous*** Rossi, 2015

Sucinlourencous adrianae Rossi, 2015

Family **Protoischnuridae** Carvalho & Lourenço, 2001

Note: This family is often questioned by neontologists or by poorly experienced paleontologists influenced by neontologists. It was already placed in synonymy before [79] but revalidated by Lourenço [51]. Doubts were, once again, raised on its validity by other authors, but based exclusively on speculation. So its validity is maintained here.

Genus ***Cretaceoushormiops*** Lourenço, 2018

Cretaceoushormiops knodeli Lourenço, 2018

Cretaceoushormiops staxi Lourenço, 2022

Genus ***Cretaceousopisthacanthus*** Lourenço, 2021

Cretaceousopisthacanthus smeelei Lourenço, 2021
